# Specific shoot formation in Miscanthus saccharif lorus (Poaceae) under different environmental factors and DNA passportization using ISSR markers

**DOI:** 10.18699/VJGB-22-04

**Published:** 2022-02

**Authors:** O.V. Dorogina, N.S. Nuzhdina, G.A. Zueva, Yu.A. Gismatulina, O.Yu. Vasilyeva

**Affiliations:** Central Siberian Botanical Garden of the Siberian Branch of the Russian Academy of Sciences, Novosibirsk, Russia; Novosibirsk State University, Novosibirsk, Russia; Central Siberian Botanical Garden of the Siberian Branch of the Russian Academy of Sciences, Novosibirsk, Russia; Central Siberian Botanical Garden of the Siberian Branch of the Russian Academy of Sciences, Novosibirsk, Russia; Institute for Problems of Chemical and Energetic Technologies of the Siberian Branch оf the Russian Academy of Sciences, Biysk, Russia; Central Siberian Botanical Garden of the Siberian Branch of the Russian Academy of Sciences, Novosibirsk, Russia

**Keywords:** Miscanthus, bioenergy, cellulose, lignin, shoot formation, DNA passportization, ISSR markers, род Miscanthus, биоэнергетика, целлюлоза, лигнин, побегообразование, паспортизация, ISSR-маркеры

## Abstract

The generic complex Miscanthus Anderss. (Poaceae) is a unique example among herbaceous plants characterized by high values of growth of aboveground vegetative mass and practical use as a valuable source of alternative energy. Miscanthus is one of the most eff icient solar energy accumulators, and since phytomeliorative use implies the cultivation of these resource plants in inconvenient and semi-shady areas, the question about the effect of insuff icient lighting on the productivity of Miscanthus arises. As a result of a long-lasing introduction effort, the Central Siberian Botanical Garden SB RAS created a population of Miscanthus saccharif lorus (Maxim.) Benth., which has good prospects for growing under the conditions of the forest-steppe area in Western Siberia. The goals of our study were: (1) to determine the peculiarities of shoot formation, (2) to assess the cellulose and lignin accumulation in M. saccharif lorus populations under different lighting conditions and (3) to perform a DNA passportization of the Miscanthus population by ISSR marking. Evaluation of shoot formation and the amount of accumulated cellulose and lignin in plants was carried out under different degrees of illumination: one variant was grown in a sunny area, and the other, in partial shade. As a result of analysis of variance, it was found that the number of shoots does not depend on environmental conditions, but on the age of the plant, while environmental conditions have a signif icant effect on plant height. Although the samples of both M. saccharif lorus variants were characterized by different rates of creation of a continuous projective cover, plants in semi-shaded areas formed up to 89.34 % of shoots compared to their peers in illuminated areas, which did not affect signif icantly the size of the aboveground mass and the cellulose content in it. As a result of ISSR-analysis of genomic DNA in the M. saccharif lorus population, unique molecular polymorphic fragments were identif ied, which can be used for identif ication and DNA passportization at the inter-population level. Thus, the complex use of M. saccharif lorus as a valuable meliorative and bioenergetic culture is due to the high adaptive potential of this species. It was found that the illumination factor has virtually no effect on the amount of the cellulose content in the shoot, and a reduced content of the technologically undesirable lignin was observed in plants growing in the partial shade conditions.

## Abstract (ru)

Уникальным примером травянистых растений, характеризующихся высокими значениями нарастания надземной вегетативной массы и практическим применением в качестве источника альтернативной энергетики, является родовой комплекс Miscanthus Anderss. (Poaceae). Мискантус относится к числу наиболее эффективных аккумуляторов солнечной энергии, и поскольку фитомелиоративное использование подразумевает выращивание этих ресурсных видов на неудобьях и полутенистых участках, то встает вопрос о влиянии недостаточного освещения на показатели продуктивности мискантуса. В результате длительной интродукции в Центральном сибирском ботаническом саду СО РАН создана перспективная для условий лесостепи Западной Сибири популяция Miscanthus saccharif lorus (Maxim.) Benth. Целью данного исследования было изучение особенностей побегообразования, оценка целлюлозы и лигнина в популяциях M. saccharif lorus при различных условиях освещенности и паспортизация перспективной популяции с помощью ISSR-маркиров. Оценка побегообразования и количества накапливаемой целлюлозы и лигнина у растений проводилась в зависимости от освещенности (один вариант выращивался на солнечном участке, а другой – в полутени). В результате дисперсионного анализа установлено, что число побегов зависит не от экологических условий, а от возраста растения, в то время как на высоту растений существенно воздействуют экологические условия. Несмотря на то что для образцов обоих вариантов M. saccharif lorus была характерна различная скорость создания сплошного проективного покрытия, растения на полутенистых участках образовывали до 89.34 % побегов в сравнении с растениями на освещенных участках, что не оказывало существенного влияния на величину надземной массы и содержание в ней целлюлозы. В результате электрофореза геномной ДНК в популяции M. saccharif lorus при амплификации с пятью ISSR-праймерами выявлены уникальные молекулярные полиморфные фрагменты, которые были применены для идентификации и паспортизации данной популяций. Таким образом, комплексное использование M. saccharif lorus в качестве средоулучшающей и биоэнергетической культуры обусловлено высоким адаптивным потенциалом вида. Обнаружено, что фактор освещенности практически не влияет на количество целлюлозы в стебле, а пониженное содержание технологически нежелательного компонента, лигнина, отмечено при выращивании в условиях полутени.

## Introduction

Over the past two decades, species of the genus Miscanthus, also known as elephant grass, have become one of the plant objects that are practically inexhaustible sources of renewable raw materials for the production of glucose, which is a basic product for many developments in the field of alternative energy (Slynko et al., 2013). Miscanthus is one of the most efficient solar energy accumulators on the planet (Dohleman, Long, 2009). According to physiological researches, Miscanthus species have high productivity potential. The production of up to 40 tons of dry biomass per hectare is associated with the C4 type of photosynthesis, which is characteristic for these species. Unlike most traditionally cultivated C4 plants, such as sugarcane and corn, Miscanthus is able to maintain a high rate of photosynthesis even under relatively low temperatures (Naidu et al., 2003; Anisimov et al., 2016). This explains the high productivity of this grass grown in more severe than natural climatic conditions for the purpose of economic use as a technical (bioenergetic) crop in the forest-steppes of Western Siberia. Phytomeliorative use implies the cultivation of a resource species in inconvenient and semi-shady areas. In particular, this also applies to M. saccharif lorus plants for photosynthesis, which requires a significant influx of photosynthetically active radiation. There is almost no data concerned with M. saccharif lorus usage as resource plants in semi-shady areas.

Currently, the problem of genetic identification of wild plant species and their populations is extremely urgent and is at the initial stage of development, although genetic passportization is believed to be an important stage required for registration and certification of new varieties (Kalayev et al., 2012).

The basis of selecting forms or varieties for the purpose of genetic passportization is to mark genetically determined characteristics using molecular methods. Some protein molecules like storage proteins or isozymes, or specific DNA loci can be used as molecular markers (Naeem et al., 2014; Chelyustnikova et al., 2019). Passportization of varieties and hybrids of many agricultural plants and crops was carried out using ISSR and IRAP markers (Sukhareva, Kuluev, 2018), as well as molecular certification of rare and endemic plant species and their natural populations like two species of Adonis, A. vernalis and A. sibirica (Boronnikova, 2009). On the basis of this technique, I.V. Boboshina, S.V. Boronnikova received a patent for the invention “Method of molecular-genetic identification of woody plant species populations” (Boboshina, Boronnikova, 2014). To identify raw materials of medicinal plants by roots or other plant tissues, DNA and chemical markers are used, since they are not tissue-specific and have a high resolution power and accuracy (Wallinger et al., 2012; Ganiea et al., 2015). However, we found no literature data on the molecular certification of promising populations and varieties of Miscanthus.

Consequently, the goals of the present study were: (1) to determine the specific shoot formation in M. saccharif lorus populations under different environment conditions, (2) to assess the cellulose and lignin accumulation in various lighting conditions and (3) DNA passportization of M. saccharif lorus population by ISSR marking

## Materials and methods

The experimental plots of the Central Siberian Botanical Garden (CSBG SB RAS, Novosibirsk, Russia) are located in the forest-steppe part of Western Siberia, which belongs to the IV lighting zone and, in terms of the total number of hours of sunshine, it is close to Krasnodar and Yalta. The object of the study was a selective population of M. saccharif lorus, identified as a result of many years of introduction experiments, which was formed from material collected in the Khasansky district of Primorsky Krai. One sample from this population was grown in partial shade (sample 1), and the other sample (sample 2) was grown in an open, well-lit area. The control (sample 3) was the introduction population of M. saccharif lorus, from which selections were made to study the features of shoot formation..

Experimental individuals were planted in 2017 on plots 2×2 m in size in four replicates. Sample 1 was placed in the penumbra (factor A0), and sample 2 – in an open, well-lit place (factor A1). On the plots, 1 rhizome (rhizome piece) with 5 shoots was planted in each of the staggered 16 holes (4 luns/m2). Thus, the total number of shoots during planting was 20 shoots/m2. Sample 3 (control) represented a continuous perennial clump located in an illuminated place. In autumn, at the end of the vegetation season, we carried out continuous pruning of the plants, leaving the height of the shoots 15 cm from the soil level

Subsequently, at the end of the growing seasons, the number of shoots was counted for the plants generated from rhizomes: for 2-year-old plants in 2018 (factor B0), and for 3-year-old plants in 2019 (factor B1). The results of the two-factor experiment were processed by the method of variance described by Dospekhov (1985).

The dynamics of growth and shooting of M. saccharif lorus was studied by the method of phenological observations, carrying out biometric measurements and counting the number of shoots formed during three growing seasons.

The chemical composition was studied in 2019 in the aerial part of the Miscanthus, cut off at a distance of 10–15 cm from the ground. Before performing the chemical analysis, the raw material was dried in air to minimize the moisture content (less or equal 8 %) and grinded up to a size of 5–10 mm. The chemical composition of plant materials was determined by standard analytical methods using the equipment of the Biysk Regional Center for Collective Use (Institute for Problems of Chemical and Energetic Technologies SB RAS, Biysk, Russia). The determination of the mass fraction of cellulose was carried out using the Kurschner method (in terms of absolutely dry raw material – a.d.m.), with the determination of the mass fraction of acid-insoluble lignin (a.d.m.), the mass fraction of pentosans (a.d.m.), ash content (a.d.m.), the mass fraction of extractives – fatty wax fraction (FWF) (extractant – dichloromethane, a.d.m.), according to the standard analysis of plant raw materials (Obolenskaya et al., 1991).

Extraction of genomic DNA from dried leaves was performed by the CTAB method (Doyle J.J., Doyle J.L., 1990). DNA concentration was determined spectrophotometrically using a BioSpectrometer kinetic and a μCuvette G1.0 microcuvette (Eppendorf, Germany).

For molecular analysis of populations, 16 ISSR (inter simple sequence repeats) oligonucleotides (primers) were tested. The most polymorphic five oligonucleotides were selected to obtain molecular genetic formulas (Table 1).

**Table 1.**
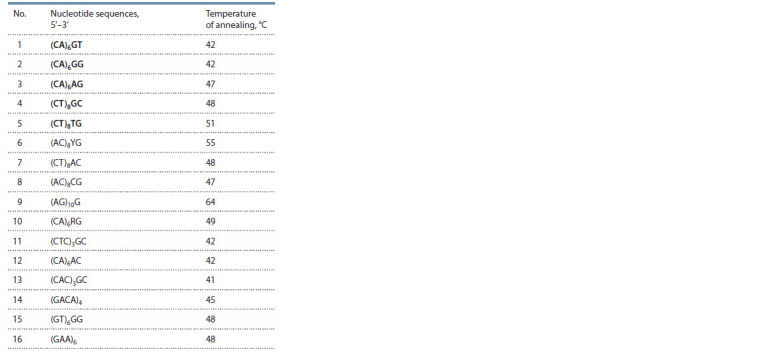
Characteristics of ISSR primers tested and selected (in bold) for the study of genetic polymorphism of M. saccharif lorus population

PCR was carried out under the following conditions: (1) DNA denaturation: 90 s at 94 °C; (2) 35 amplification cycles: 40 s at 94 °C, 45 s at 41–56 °C (primer annealing) and 90 s at 72 °C; (3) elongation: 5 min at 72 °C. The PCR mixture with a volume of 25 μL consisted of 2.7 mM MgCl2, 1.25 mM primer, 0.4 mM dNTPs, 2.5 μL 10× PCR buffer, 1 unit of Taq DNA polymerase and 20 ng genomic DNA. PCR was performed on a Thermal Cycler C1000 amplifier (Bio-Rad, USA). Electrophoretic analysis of ISSR-PCR products was carried out in 1 % agarose gel. The amplified fragments were stained with SYBR-Green (ThermoFischer Scientific). Visualization and recording of the separated PCR fragments was carried out using the Gel-Doc XR+ gel documentation system and the ImageLab Software Imaging System (Bio-Rad).

Molecular genetic formulas for the passportization of the M. saccharif lorus population were drawn up according to the principle proposed by A.A. Novikova and co-authors (Novikova et al., 2012). Statistical analysis was carried out using the MS Excel program.

## Results

**Fig. 1.**
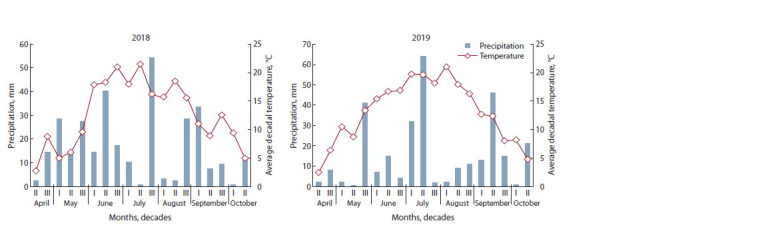
Hydrothermal conditions of the growing seasons 2018–2019.

Under experimental conditions, M. saccharif lorus plants regrowth and further development was observed in the third decade of May – first decade of June, 2018. No active growth of the vegetative mass was noted in the third decade of May since the air temperature did not exceed 9.6 °C (Fig. 1). Starting from the second decade of June, with an increase in temperature, the number of shoots rose due to active tillering and intensive growth rates.

From the meteorological point of view, 2019 was favorable for the elephant grass. The average temperature in May was 10.8 °С, which contributed to active vegetation. Further, in plants with a well-developed and successfully overwintered underground shoot system, an aboveground part was formed, resembling a clearly polycentric biomorph: diasporas are formed on the plagiotropic shoots of this species, at the moment of the appearance of their own root system they are fixed, maintaining a connection with the mother plant.

In June, the temperature slowly increased without drops (see Fig. 1), the tillering process took place from July (especially during the period of maximum precipitation) to the beginning of August. Plants in all variants formed a greater number of shoots during the 2019 growing season than in 2018. In the second half of August, the activity of the tillering process correlated with the temperature and humidity level (22 mm of precipitation – 33 % of the norm). Plants reduced the productivity of the vegetative mass and started generative processes. At this time, active elongation of shoots was observed due to the increase in a hollow peduncle, the formation of an upper “flag” leaf and the appearance of panicle inflorescences, which lead to growth of the vegetative mass of shoots.

To identify the effect of ecological conditions and plant age on the shoot formation of M. saccharif lorus (in 2018 and 2019), a two-factor analysis of variance was carried out (Tables 2 and 3). As can be seen from Table 2, the number of formed shoots was significantly influenced by the age of rhizomes (B); at the same time, the influences of environmental conditions (A) and the interaction of factors (AB) were insignificant. The factor of ecological conditions (A) has an influence on plant height (see Table 3), but the effects of the age of rhizomes (B) and the interaction of factors (AB) were insignificant

**Table 2.**
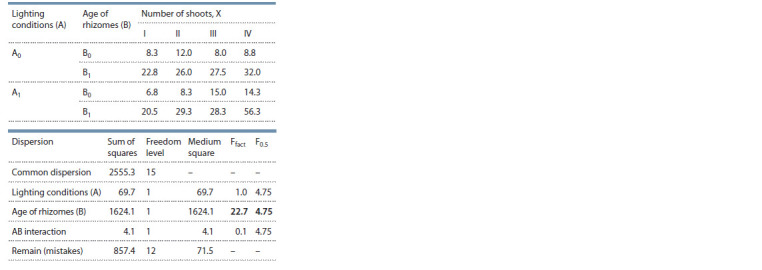
Results of the variance analysis of a two-factor experiment to study the inf luence of environmental conditions and the age of rhizomes on the number of shoots of M. saccharif lorus

**Table 3.**
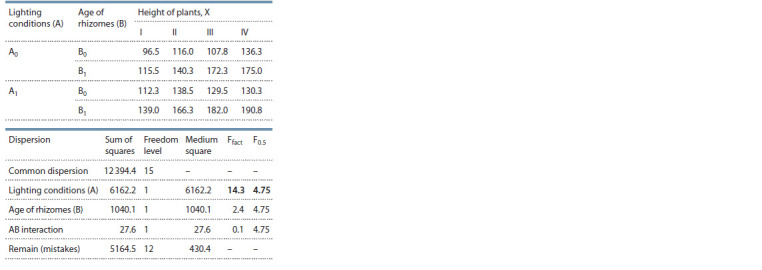
Results of the variance analysis of a two-factor experiment studying the inf luence of environmental conditions and the age of rhizomes on the height of M. saccharif lorus plants

Thus, as a result of a variance analysis (see Table 2, at F = 0.5), it was revealed that the factor of the age of rhizomes (B 22.7 > 4.75) has a significant effect on the number of shoots, but not the ecological conditions. Meanwhile, the height of the plants largely depends on environmental conditions (A 14.3 > 4.75), but not on the age (see Table 3).

**Fig. 2.**
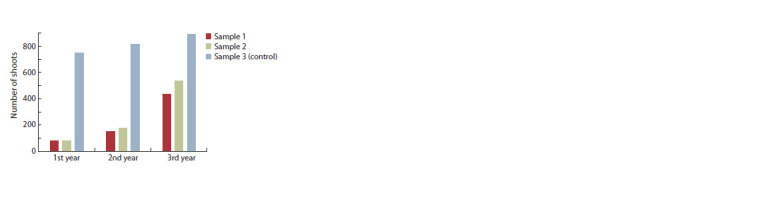
Features of shoot formation of M. saccharif lorus specimens under various ecological conditions. Sample 1 – semi-shade area, sample 2 – well-lighted area, sample 3 (control) – perennial plants.

The control population consisted of perennial plants located in a well-lighted place. As is shown on Figure 2, there is a little increase in the number of shoots in control plants that have previously formed a dense curtain, and, on the contrary, intensive shoot formation in young plants (initially represented by rhizomes) at the 2nd and 3rd years of life. It was revealed that M. sacchariflorus plants in semi-shady areas at 2 years of age formed up to 89.34 % of shoots in comparison with illuminated areas

However, sample 1, which has grown in the shade, in terms of the number of shoots developed during the second (148 shoots) and third (433) years of life, lags behind sample 2 with 177 and 537 shoots, respectively. The effect of higher illumination stimulates tillering in sample 2, which leads to quick closing of separately located plants.

Less intense tillering was observed in sample 1 in the penumbra; however, in this case, too, the projective vegetation cover in the third year of experiment was quite high – from 65 to 75 %. Sample 3 has been growing in one place for more than 15 years. It is noted that plants actively grew and developed annually, no degradation phenomena were observed. The projective cover was over 70 %. However, the possibilities of intensive shoot formation were practically exhausted, therefore, in 2019, the increase in the number of shoots compared to 2018 was only 9.06 % (816 and 890 shoots). For sample 1 located in the semi-shade area, this increase was 192.57 % (148 and 433), and for sample 2 located in the well-lighted area – 203.40 % (177 and 537).

Chemical analysis of these three samples, carried out on the material of M. saccharif lorus in 2019, separately on the stems (since the stem cellulose is valued higher) and leaves, showed that the cellulose content in the penumbra (50.9 %) was higher than in the illuminated area (50.1 %). Reduced (by 12 %) content of a technologically undesirable component lignin was noted in the least economically valuable semishady area (Table 4). This could be caused by the fact that tissue differentiation of shoots, including lignification, occurs more intensively in sufficient illumination.

**Table 4.**
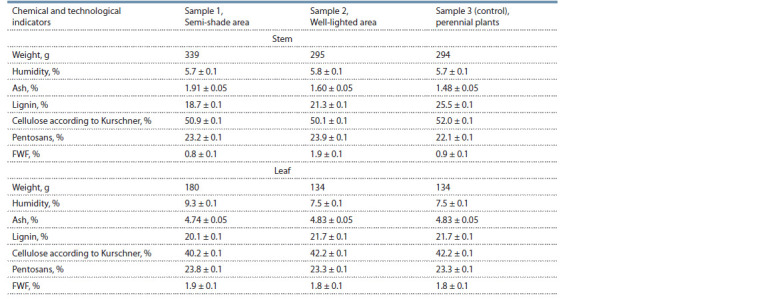
Chemical composition of three samples of M. saccharif lorus in 2019 Notе. FWF – fatty wax fraction; the half-width of the conf idence interval was determined at the signif icance level of 0.05.

The mass fraction of cellulose in the leaf regardless of the light conditions (40.2 % in the partial shade area and 42.2 % in the sunny area) is significantly lower than in the stem, which is in good agreement with the previously obtained results (Gismatulina et al., 2019). Similarly to the stem, the mass fraction of lignin is 7.6 % lower in the partial shade area than in the sunny area. The mass fractions of pentosans, FWF, and ash are approximately at the same level both in the stem and in the leaf, regardless of the lighting conditions of the plantation.

**Fig. 3.**
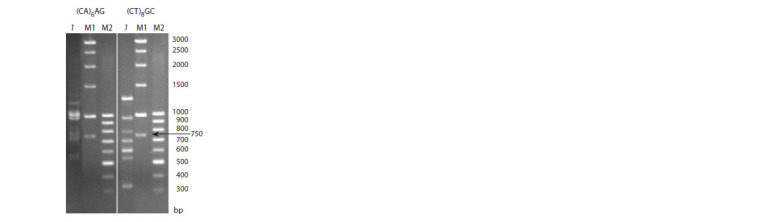
PCR-ISSR prof ile of M. saccharif lorus generated from amplif ication with the primers (CA)6AG and (CT)8GC. The track numbers correspond to the samples: 1 – M. saccharif lorus (No. 1), M1 and M2 – molecular weight markers.

As a result of electrophoresis of PCR products of genomic DNA in the M. saccharif lorus population obtained by amplification with five selected ISSR primers, a high genetic polymorphism of the studied objects was found (Fig. 3).

From one to four specific molecular markers (unique PCR fragments) were identified (Table 5). As follows from Table 5, the length of polymorphic fragments in ISSR analysis ranged from 660 to 2000 bp. The identified unique molecular polymorphic fragments representing sequences of a certain length were selected for passportization of M. saccharif lorus population.

**Table 5.**
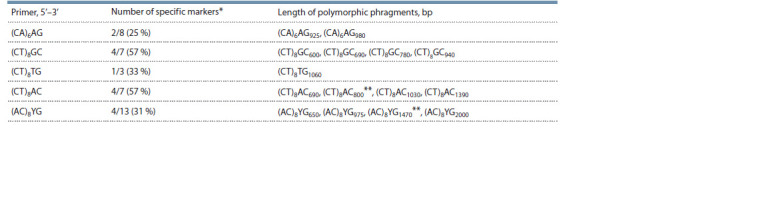
ISSR markers used in the study to identify the molecular formula of M. saccharif lorus population * Total number of markers (denominator), number of unique markers (numerator) and their percentage (in brackets). ** ISSR markers with a weak f luorescent signal.

Thus, taking into account the genetic formula proposed by A.A. Novikova with co-authors for Rhododendron canadense (Novikova et al., 2012), the genetic formula for the M. saccharif lorus population will be the following: ISSR/(CA)6AG-925,980/(CT)8GC-600,690,780,940/ (CT)8TG-1060/(CT)8AC- 6 9 0 , 8 0 0 , 1 0 3 0 , 1 3 9 0 / (AC)8YG-650,975,1470,2000.

## Discussion

The study of the specificity of shoot formation in M. saccharif lorus introduced into CSBG under the conditions of the continental climate of Western Siberia showed that early generative development of plants is undesirable for growing this species as a bioenergetic culture, since the accumulation of biomass stops. It was found that this species has a rather long period of active growth. It should be taken into account that plants of M. saccharif lorus start growing only after the air warms up to 25 °C. In experimental 3-year-old plants the projective vegetation cover in triplicate varied from 70 to 80 %

Based on the results of variance analysis we can conclude that the number of shoots depends on the age of the plants and the influence of environmental conditions, and the interaction of these factors on the number of shoots is insignificant. At the same time, ecological conditions have a significant effect on plant height and age, and the interaction of these factors practically do not affect plant height. In this regard, an important issue in the study of the shoot formation of M. saccharif lorus is the initiation of tillering period, which is associated either with the beginning of the growth of lateral shoots in the zone of shortened internodes (Langer, 1963; Smelov, 1966), or with intensive growth of this zone (Dobrynin, 1969; Gorchakova, 2003).

It should be noted that the mass fraction in the stem of the technologically significant component cellulose does not change depending on the lightning conditions (50.9 % in partial shade area and 50.1 % in the open area). At the same time, the mass fraction of lignin, which adversely affects technological processes, was 12 % lower in the penumbra.

Thus, it was found that the adaptive potential of M. saccharif lorus, the high content of cellulose (52.04 %) with a relatively low content of lignin (21.3 %) allows to suggest the population as an environment-improving and bioenergetic culture.

For genetic passportization of the population, five ISSR markers were selected. Based on our studies and the results obtained by other authors, we can conclude that ISSR primers which have been used are polymorphic and can be recommended for identifying other samples, populations and species, as well as for composing genetic formulas and passports for the genus Miscanthus (Boronnikova, 2009; Artyukhova et al., 2011; Novikova et al., 2012; Lebedev et al., 2014). I.A. Klimenko with co-authors carried out identification and certification clover varieties using SSR and SRAP markers and proposed a set of DNA markers (Klimenko et al., 2020). The data obtained using DNA analysis are the most objective for describing plant varieties and species, since they are not susceptible to genotypic variability and mostly have a codominant type of inheritance (Ramazanova, Kolomytseva, 2020).

The genetic passport of M. saccharif lorus, presented as a genetic formula generated by amplified DNA, contains information about the method used, oligonucleotide sequences, and specific amplified DNA fragments lengths. If necessary, it is possible to improve the form of recording the molecular genetic formula indicating the specificity level of a fragment (generic, species, polymorphic), as it was suggested by S.V. Boronnikova (2009). In general, the molecular genetic formula population makes it possible to determine the belonging of the Miscanthus individuals not only to the species and variety, but also to a specific population.

## Conclusion

The results obtained during the study allow concluding that M. saccharif lorus can be successfully grown in semi-shady forest steppes of Western Siberia, and the lignin content in raw plant material will be reduced by the time of harvesting in case of growing at the local microecological conditions.

The high projective vegetation cover under various environmental conditions, as well as the longevity of the clones, indicate the prospects for the phytomeliorative use of selected forms of M. saccharif lorus in the continental climate of the forest-steppe of Western Siberia. The content of cellulose in the stem, the most important component in technical plant material, varies slightly depending on the lighting conditions. At the same time, the content of lignin, which negatively affects technological processes, turned out to be lower in plants grown in partial shade.

The obtained molecular genetic formulas for the M. saccharif lorus population make it possible to determine the belonging of individuals of Miscanthus not only to the species and variety, but also to a specific population.

Genetic passportization based on molecular data of promising forms of Miscanthus, the development of scientific and practical recommendations and a set of cultivation techniques will make it possible to use the representatives of this genus in breeding under the conditions of the continental climate of Western Siberia.

The selective work with the varieties obtained by vegetative reproduction of the perspective individuals and their molecular DNA identification make it possible to recommend the genus Miscanthus as an environmentally friendly technical crop and as a renewable source of plant material promising for the implementation of an alternative bioenergy program in Western Siberia.

## Conflict of interest

The authors declare no conflict of interest.
